# Ecological network analysis of traits reveals variable response capacity to stress

**DOI:** 10.1098/rspb.2023.0403

**Published:** 2023-05-10

**Authors:** Rebecca V. Gladstone-Gallagher, Judi E. Hewitt, Ewa Siwicka, Johanna M. Gammal, Marco C. Brustolin, Alf Norkko, Conrad A. Pilditch, Simon F. Thrush

**Affiliations:** ^1^ University of Auckland, Auckland, New Zealand; ^2^ Tvärminne Zoological Station, University of Helsinki, Hanko, Finland; ^3^ University of Waikato, Hamilton, New Zealand; ^4^ Institute of Marine Research (IMR), Arendal, Norway

**Keywords:** cumulative effects, resilience, benthic ecology, recovery, multiple stressors, response diversity

## Abstract

Response diversity increases the potential ‘options’ for ecological communities to respond to stress (i.e. response capacity). An indicator of community response diversity is the diversity of different traits associated with their capacity to be resistant to stress, to recover and to regulate ecosystem functions. We conducted a network analysis of traits using benthic macroinvertebrate community data from a large-scale field experiment to explore the loss of response diversity along environmental gradients. We elevated sediment nutrient concentrations (a process that occurs with eutrophication) at 24 sites (in 15 estuaries) with varying environmental conditions (water column turbidity and sediment properties). Macroinvertebrate community response capacity to nutrient stress was dependent on the baseline trait network complexity in the ambient community (i.e. non-enriched sediments). The greater the complexity of the baseline network, the less variable the network response to nutrient stress was; in contrast, more variable responses to nutrient stress occurred with simpler networks. Thus, stressors or environmental variables that shift baseline network complexity also shift the capacity for these ecosystems to respond to additional stressors. Empirical studies that explore the mechanisms responsible for loss of resilience are essential to inform our ability to predict changes in ecological states.

## 1. Background

Globally, irreversible degradation of ecosystem functions and services has been associated with the loss of species and habitat diversity [1–4]. As the number and intensity of environmental stressors increases, ecosystems lose the capacity to cope with additional stress. This loss of response capacity is nonlinear and results in rapid (and often irreversible) changes in ecosystem state when tipping points are crossed [3,5]. Limiting irreversible damage requires actions that enhance ecological resilience, but this necessitates a deep understanding of the mechanisms that underpin its loss. Such understanding can be gleaned and improved from empirical assessments of how response capacity shifts with environmental stressors and simultaneously shed light on the mechanisms responsible for the erosion of resilience. Here, we use biodiversity response data from a large-scale marine soft-sediment field experiment to explore how and where along environmental gradients ecosystems lose response capacity to stress.

The general consensus from decades of focused research is that biodiversity underpins ecosystem function and stability [6–8]. While establishing relationships between species richness, ecosystem functioning and stability have been useful in understanding the impacts of biodiversity loss [7,8], it is now essential we develop mechanistic understandings of how this loss affects ecosystem transitions to enable more accurate predictions of tipping points. To this end, functional rather than species diversity has been used (i.e. usually using species biological traits as proxies for function) [9–12]. It has been argued that functional diversity is more relevant to ecosystem functioning because it accounts for the level of redundancy inherent in most species rich ecosystems (i.e. a loss of species does not necessarily result in a loss of function because multiple species can underpin similar functions [13–15]. The functional redundancy concept has been expanded further conceptually to consider ‘response diversity’. Response diversity is a measure of how functionally similar species can exhibit a range of different response strategies to stress (derived from species ‘response traits’) and this diversity increases response capacity [5,16,17]. Despite these conceptual advancements, there are very few empirical demonstrations of the linkages between response diversity and stability that are critical to providing a mechanistic understanding of real-world ecosystem resilience [5,14].

There are a variety of ways to assess community response diversity. It is common to assign biological traits of species or populations as proxies for their effect on the ecosystem function (effect traits) or their ability to respond to stress (response trait) [11]. This categorization enables analyses to be conducted using widely gathered community data without the need for specific, often difficult to collect, functional process-based measurements enabling broader ecological questions to be answered (although this approach is not without criticism; e.g. see [18]). While measures of trait diversity have been useful in elucidating changes across environmental gradients, they often collapse information into indices (e.g. Shannon Weiner, *F*_ric_, *F*_dis_, *F*_eve_) [19,20], potentially simplifying the nuances of ecological responses that are characterized by many interacting variables.

Recently, stress responses in trait diversity have been assessed through changes in trait networks (‘network analysis of traits'; NAT) where the concepts of social network analysis are applied to ecological communities based on their characterized traits [21]. Investigating changes in communities using a network approach is useful in the context of resilience as networks have the ability to show relational changes of the whole community network while maintaining a high level of detail into interactions. This approach is intuitive in the context of resilience thinking because in many applications resilient and stable networks are often characterized by higher connectance and complexity [21,22]. Previous network analysis of traits has explored functional redundancy of species that overlap and are connected by similar functional trait compositions [21]. When examining functional redundancy, networks are set up with species as nodes and trait similarity defines connections. However, to explore response diversity, here we are interested in trait networks that examine trait cooccurrence among ecosystem regulating traits and resilience traits. That is, the more connections a community has between ecosystem regulating traits and resilience trait nodes (i.e. response diversity), the more ‘options’ the community has for responding to stress without losing ecosystem function (i.e. response capacity). Analysing changes in network architecture related to the number and strength of connections may also assist in linking empirical research on response diversity to theory of the different elements of ecosystem stability and resilience [23].

Here, we use data from a nationwide field experiment and a network analysis of traits to explore how the response diversity (indicated by network complexity) of soft-sediment macroinvertebrate communities is linked to response capacity to stress. Our experiment occurred at 24 intertidal sites in 15 estuaries across New Zealand, and at each site, we experimentally elevated porewater nutrient concentrations (a common stressor associated with eutrophication in coastal systems globally). The sites were chosen to vary in water column turbidity, an environmental characteristic that is implicated in variation in soft-sediment ecosystem biodiversity, functions and processes [24]. Importantly, the wide range of sites supported a gradient of natural variation in macroinvertebrate trait network architectures (from very simple to complex networks) that we could use to explore (1) how natural variability in network architecture (response diversity) was related to resilience to an added nutrient stressor (response capacity), and (2) how the added stressor eroded response diversity through changes in the trait networks. We hypothesized that as communities become stressed by added nutrients, there would be a loss of connections between functional and resilience traits (i.e. an erosion of response diversity), and we expected that the initially more complex networks would be more resilient and less prone to connection losses with nutrient stress than simpler ones. Such empirical demonstration of the erosion of resilience is critical to explore the context dependencies in responses to stressors and the breakdown of functional performance and response capacity that occurs prior to tipping points.

## 2. Methods

### (a) Experimental design

The data used in this paper came from a large field experiment described in detail in Thrush *et al*. [25] and Gammal *et al*. [26]. Briefly, the 24 mid-intertidal sites occurred across a gradient in water column turbidity (environmental variables including sediment grain size, organic and nutrient content also varied across the sites; see [26]), but all sites were occupied by the biomass dominant bivalves, the suspension-feeding clam *Austrovenus stutchburyi* and deposit-feeding wedge shell *Macomona liliana.* At each site, a nutrient enrichment manipulation was set up by establishing nine 9 m^2^ square plots (marked with 4 small corner pegs) that were randomly assigned to three treatments (i.e. *n* = 3 plots per treatment): high nutrient (nitrogen) addition (where 600 g N m^−2^ was added), medium nutrient addition (300 g N m^−2^), and disturbance control (no added N). In the high and medium nutrient treatments, we injected Nutricote slow release urea fertilizer at regular intervals, at a depth of 15 cm (40–0–0 N:P:K), elevating the porewater nitrogen concentrations for an extended period. The elevation of porewater N concentration aimed to simulate the chronic effects of eutrophication stress in estuaries (a key stressor for estuaries globally), but it does not simulate the water column effects of eutrophication [27]. Previous experiments have demonstrated these levels of enrichment reduce macrofaunal species richness but do not defaunate sediments [27,28].

The experiment was left for seven months before sampling to allow the nutrients to diffuse through sediments and the ecosystem to respond to the enrichment [25,27,29]. During this time an Odyssey PAR logger deployed 10 cm off the seabed at each site recorded (at 10 min intervals) photosynthetically active radiation (PAR) reaching the seabed (giving an ecologically relevant proxy for water column turbidity) [30]. Because our systems are relatively oligotrophic [27,31], PAR provides a proxy for suspended sediment-based water column turbidity. We averaged daily PAR readings during daytime emersion periods (±2 h of high tide), then averaged these to estimate mean daily PAR. After seven months we collected five sediment cores (2.6 cm dia. × 2 cm depth) from each plot which were pooled, then frozen and later subsampled for measurements of sediment grain size, organic content and chlorophyll *a* content using standard methods [25,32]. An additional five sediment cores were collected at depths of 5–7 cm and 0–2 cm in the sediment for measurement of sediment porewater nitrogen concentration. These cores were centrifuged immediately to remove the porewater from sediment, and then the water filtered on a Whatman GF/C fibre filtre and frozen for later analysis of NH_4_^+^ concentration (because the added urea fertilizer hydrolyses to ammonium). At this time, two macrofauna cores (13 cm dia. × 15 cm depth) were also taken and pooled from each plot. These were sieved on a 500 µm mesh sieve and preserved in 70% Isopropyl alcohol awaiting identification of macrofauna taxa to the lowest possible taxonomic level (usually species) using a stereo microscope.

### (b) Assigning traits

We assigned traits to all macrofauna taxa based on their ability to influence three ecological dynamics: (1) ecosystem regulation (analogous to ‘effect’ traits); (2) recovery from disturbance; and (3) resistance to stress (the latter two both constitute ‘response’ traits). The ecosystem regulating traits (REG) refer to those that influence how the ecosystem functions. Included are traits that alter sediment biogeochemistry, nutrient cycling, primary and secondary production (e.g. sediment mixing, feeding mode), habitat stability (e.g. sediment topographic changes), as well as those that influence the rates of these processes (e.g. mobility, size) [9]. Recovery traits (REC) are individual and population level traits that influence recovery potential following disturbance. These include those associated with the taxa's life-history and mobility strategies (e.g. size, larval, juvenile and adult mobility), as well as those that consider how population characteristics (time to maturity, and numerical dominance and occurrence) influence the taxa's ability to colonize and establish following disturbance [33]. Resistance traits (RES) are those that are generically associated with the taxa's resistance to a range of stressors and disturbances (e.g. predators usually are enhanced by disturbance, surface dwelling animals are more likely to be exposed to stress). The REC and RES traits are not specific to the manipulated stressor (nutrient enrichment) and are instead generically related to the ecosystem's capacity to respond to a wide range of additional stressors (e.g. sedimentation, physical disturbance). This context allows a more general view of how environmental gradients and added stressors alter response diversity which is important in driving response capacity to a range of stressors or disturbances. Characterizing the networks by the interrelationships among REG, RES and REC traits allows us to determine how stressors will affect the resilience of ecosystem functions. RES and REC traits were defined as either inhibiting (indicated by REC_i_ and RES_i_) or enhancing (indicated by REC_e_ and RES_e_) resistance and recovery. Full explanation of methods for assigning traits along with a table of the traits are given in electronic supplementary material, S1.

### (c) Network analysis of traits (NAT)

To tease apart trait network responses to nutrient enrichment across the 24 sites, a network was built for each site (*n* = 24) and enrichment treatment (*n* = 3; i.e. *n* = 72 networks total). In the networks, each node was a trait modality, and the number and strength of the connections between each trait node were established using the *cooccur* function in R [34]. *Cooccur* uses a probabilistic approach to show the most significant pairs of traits. When a threshold is enabled, it filters out trait pairs that are expected to have less than one cooccurrence together. This approach is relevant to assessments of response diversity because if a trait pair only occurs in one species, there is no response diversity. Thus, *cooccur* was used as a filter to remove trait pairs from the subsequent network analysis that probabilistically had no response diversity. Following the *cooccur* procedure, we used the observed number of species having both traits to characterize the strength of the connections in the network, as this directly relates to response diversity. That is, if an important REG trait is connected to many different REC and RES traits then there are more ‘options’ for that regulating trait to resist or recover from stress.

For each site-treatment, we built a network in *Gephi* using the Fruchterman-Reingold layout algorithm (a two-dimensional, force-directed network transformation). In this form, the connections between nodes act like springs and the nodes are attracted to each other or pushed apart by the strength of the connections they have with other nodes (described in [21]). The connections in the network are the number of species that share the trait pair and so trait pairs (nodes) with higher numbers of species sharing them are more attracted to one another and appear closer in the network. Trait pairs with low numbers of species connecting them (or low response diversity) appear further away in the network, as do nodes that are less connected to other nodes.

**Figure 1.**
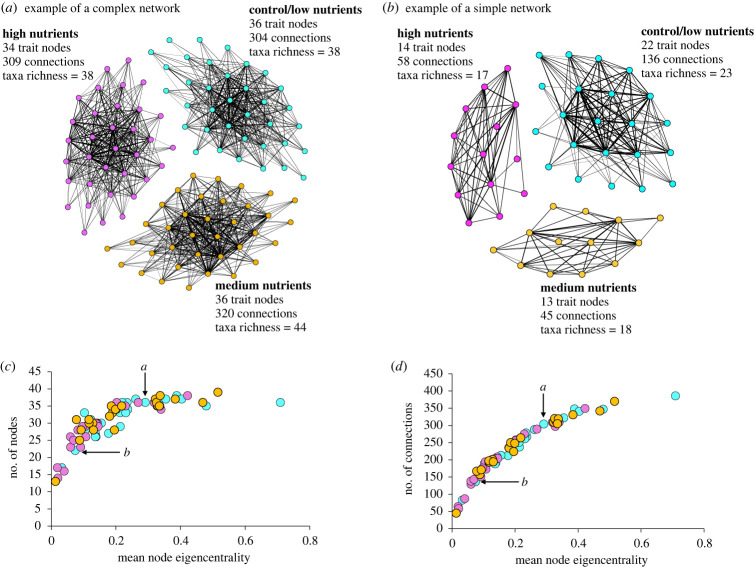
Examples of trait networks. (*a*) A complex network at a site where nutrient enrichment (medium nutrients = yellow, high nutrients = pink) did not alter network architecture compared to controls (blue). (*b*) A relatively simple network at a site where nutrient enrichment resulted in a loss of trait nodes (medium nutrients = yellow, high nutrients = pink) and connections among trait pairs compared to the control community (blue). Trait network diagrams for all sites are shown in electronic supplementary material S2. (*c*,*d*) The relationship between the mean node eigencentrality and the number of (*c*) nodes and (*d*) connections for each network is shown. The location of the control networks depicted in (*a*) and (*b*) are indicated by the arrows.

*Gephi* provides a number of network metrics (e.g. [21]; electronic supplementary material S2), many of which are highly correlated (e.g. figure 1*c,d*) and are either not intuitive nor related to our hypothesis. We selected two that were relevant to our hypothesis: the total number of nodes and the total number of connections. We also derived three more relevant metrics (table 1): the number of trait connections between (1) regulating and recovery enhancing nodes (REG∼RECe), and (2) regulating and resistance enhancing nodes (REG∼RESe), as well as (3) the mean node eigencentrality. *Gephi* gives each node in the network an eigencentrality score based on its connectance to other highly connected nodes, indicating the node's relative influence in the network [35]. Networks with high mean node eigencentrality thus have high connectance and many nodes that are ‘central’ to the structure of the network (e.g. compared visually in figure 1). Because eigencentrality is a relative measure (i.e. a node's connectance is relative to all other nodes in the network), individual node scores were obtained from a network analysis based on all sites and treatment data. We then calculated the mean node eigencentrality for each site-treatment network, and as it is a relative measure across sites and treatments, we used it as a proxy for ‘network complexity’ (figure 1*c,d* show how mean node eigencentrality co-varies with other network metrics that collectively explain network complexity).

**Table 1.** Metrics used to assess nutrient enrichment effects on trait network architecture. All metrics except mean node eigencentrality were based on individual site-treatment networks. Because node eigencentrality is a relative measure of a nodes' connectance relative to all other nodes in the network, this was obtained from a network that had all sites and treatment clusters in it (see Methods section).

network metric	relationship to response capacity
mean node eigencentrality	a node with a high eigencentrality is one that is connected to other nodes that have high connectance, and so the mean node eigencentrality gives a relative proxy for network complexity; here, we expect that high complexity is related to greater response capacity
total number of trait modalities (i.e. nodes)	a loss of trait nodes signals less trait diversity and therefore less response capacity
total number of trait connections (i.e. number of species that share pairs of traits)	a loss of connections with stress signals a loss of response capacity
number of trait connections between regulating and resistance and recovery enhancing modalities i.e. REG∼RECe, REG∼RESe (the subscripted e indicates an enhancing trait as opposed to an inhibiting one)	a loss of connections between ecosystem regulating trait modalities and resilience trait modalities signals a loss of stress response capacity of the ecosystem and potentially lowered functional performance

### (d) Statistical analysis

We were interested in how trait network architecture varied across our 24 sites, because we hypothesized that this natural variation may influence the site specific response to nutrient enrichment. To visualize the natural variation in network complexity, we plotted mean node eigencentrality in the control plots against two environmental variables of interest in this study: water column turbidity (a planned source of site-to-site environmental variation) and porewater nutrient concentration (a variable we experimentally manipulated within each site but that could also be expected to vary across the sites). These biplots confirmed that our site selection spanned a gradient of natural variation in network complexity (i.e. response diversity; see Results).

We then explored how the different trait networks (as measured by number of trait nodes, number of connections, and the number of REG∼RECe and REG∼RESe connections) responded to nutrient enrichment and varied as a function of the baseline network complexity (i.e. the mean node eigencentrality in the controls, hereafter referred to as ‘baseline network complexity’). An initial visual inspection revealed a range of responses to nutrient enrichment, from no response (e.g. figure 1*a*), to a large loss of trait pair connections and nodes with nutrient addition (e.g. figure 1*b*), and sometimes medium levels of nutrients increased the number of connections (see Results). To remove the site-based variation in network metrics which may mask nutrient treatment effects, we focused on the nutrient treatment effect-size for each metric (i.e. the nutrient treatment value divided by the control value). Then, *t*-tests were used to test for nutrient treatment effects. The network metrics for medium and high enrichment effect-size were tested for differences from control values using one-sample *t*-test and between enrichment levels with a two-sample *t*-test.

Finally, we explored the relationship between nutrient treatment effect-size and the baseline network complexity. We used quantile regression (at the 10th and 90th quantile, and every 10th quantile in between) to visually assess how the variance in treatment effect-size for the network metrics changed with baseline network complexity. All analyses were performed in Rstudio and the data used in this manuscript are available from the Dryad Digital Repository [36].

## 3. Results

### (a) Variation in site network complexity across the 24 sites

**Figure 2.**
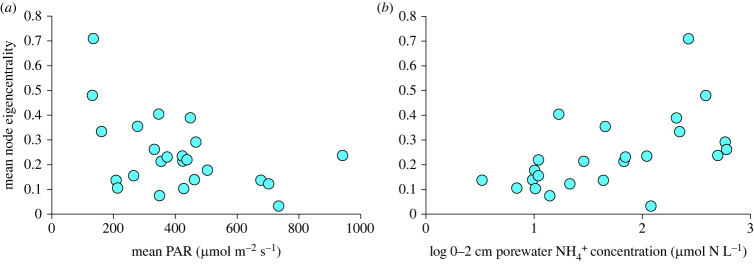
Network complexity (using mean node eigencentrality as a proxy statistic for complexity) in control trait networks as a function of (*a*) mean photosynthetically active radiation (PAR; a proxy for water column turbidity) and (*b*) porewater NH_4_^+^ concentration in the surface sediments (0–2 cm). Porewater nutrient concentrations are shown on a log scale.

We found that across the 24 sites, there was natural variation in baseline network complexity. The baseline network complexity (i.e. mean node eigencentrality in controls) ranged from 0.01 to 0.71 and was somewhat related to the variability in Mean PAR and surface porewater nutrient concentrations (figure 2). Baseline network complexity was generally higher with higher water column turbidity (i.e. decreased with increasing mean PAR; figure 2*a*) and porewater ammonium concentration (figure 2*b*).

### (b) Trait network responses to nutrient enrichment

**Figure 3.**
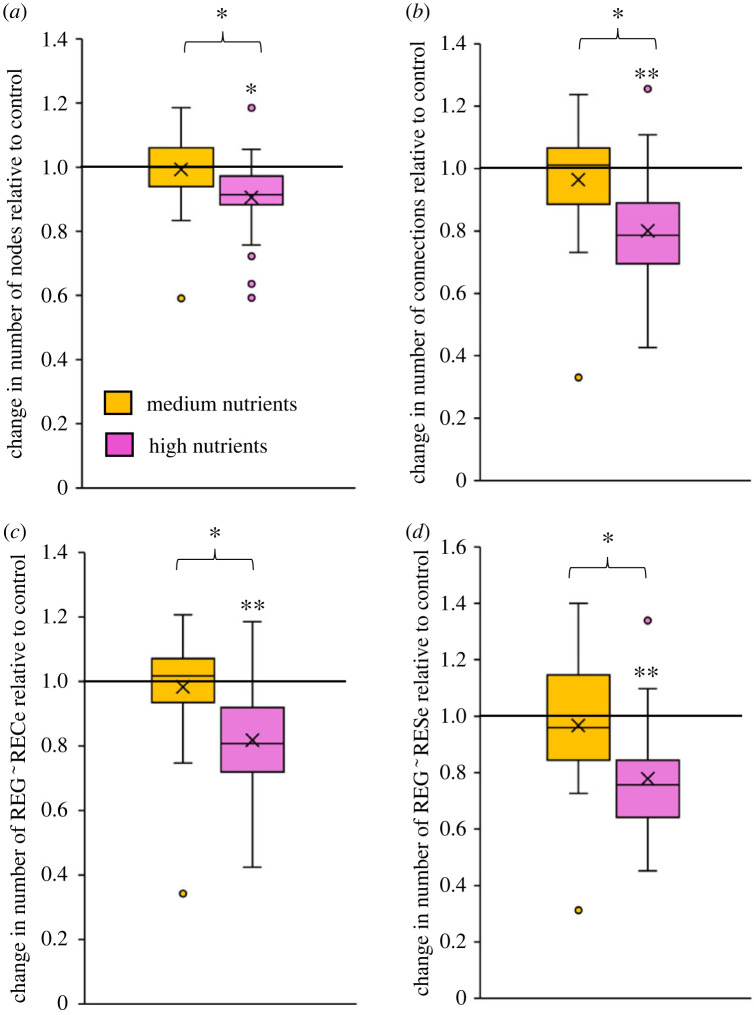
Box and whisker plots showing the nutrient treatment effect-size as the change in the number of (*a*) trait nodes, (*b*) total connections, (*c*) number of REG∼RECe connections and (*d*) REG∼RESe connections in medium (orange) and high (pink) nutrient treatments. A value above 1 means the network variable increased relative to the control, whereas below 1 the network variable decreased. The asterisk above an individual treatment box indicates the treatment is significantly different from 1 (i.e. control) and an asterisk above both boxes indicates the medium and high treatments were significantly different from each other in their effect size. *significance level *p* < 0.02; **significance level *p* < 0.0001 (full statistical results shown in electronic supplementary material S2). The whiskers represent the max and min (excluding outliers), the box denotes the 25th percentile, the median and the 75th percentile, and the mean is marked with a cross.

There was variability in nutrient treatment effect-size across the sites, but overall the high nutrient treatment resulted in a significant reduction (by up to > 50%) in all network metrics relative to controls (i.e. values less than 1 indicate a reduction relative to controls; figure 3) (*t*-test, *p* = 0.00002–0.002; electronic supplementary material S2, table S2.2). Patterns were relatively consistent across the different network metrics (figure 3). In the medium nutrient treatment, network metrics were more variable among sites, and there were both positive (i.e. enhanced networks relative to controls) and negative effects on the network (figure 3). For all network metrics, the effect-size was significantly different between the medium and high treatments (*t*-test, *p* = 0.003–0.02; table S2.2 in electronic supplementary material, S2).

**Figure 4.**
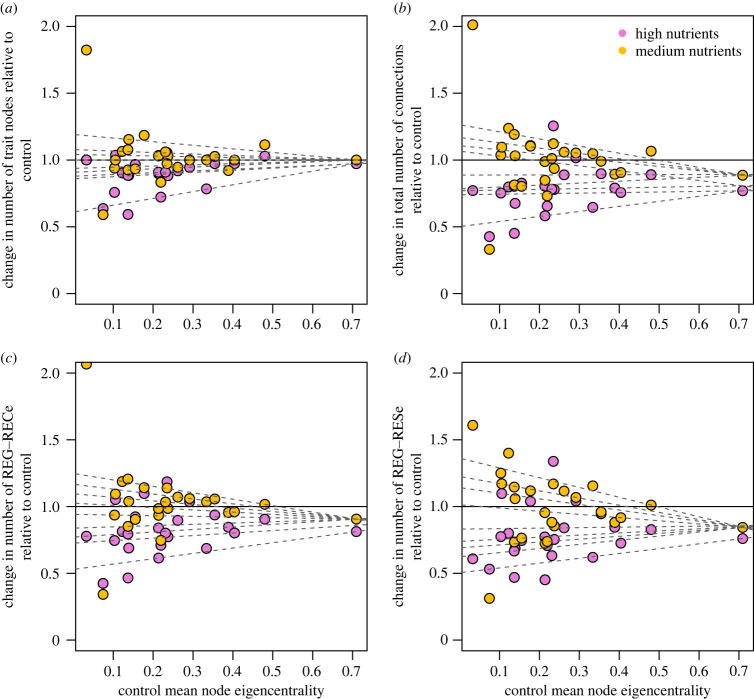
Nutrient treatment effect-size shown by the change in the number of (*a*) trait nodes, (*b*) total connections, (*c*) number of REG∼RECe connections, and (*d*) REG∼RESe connections relative to the control plots in medium (orange) and high (pink) nutrient treatments as a function of the baseline network complexity (i.e. the mean control node eigencentrality). A value higher than 1 on the y axis means the nutrient enriched community gained connections or nodes in the trait network relative to the controls at the site, while a value less than 1 means the nutrient treatment resulted in a loss of connections or nodes. Dashed lines are fitted quantile regressions between the 10th and 90th quantiles (including every 10th quantile in between).

The nutrient enrichment effect-size varied across the sites, and this was related to our measure of baseline network complexity. As this network complexity increases, the variability in nutrient enrichment effect-size decreases (depicted by the converging quantile regression lines in figure 4). Visually the lower quantile lines are largely driven by the response to high nutrients (i.e. pink datapoints in figure 4) and the upper quantile lines are driven by the response to medium nutrients (i.e. orange datapoints in figure 4).

## 4. Discussion

Our study using resilience trait networks found that seafloor communities exhibited different trait network architectures, from very simple networks made up of small numbers of trait nodes, and few connections between them, to highly complex networks characterized by high numbers of trait nodes and many connections among them. Because our networks are built with traits that are related to ecosystem regulation, recovery and resistance to stress, theoretically, the more complex the network, the higher the diversity of response strategies in the community. We tested this hypothesis with our field experiment, which showed that the changes in architecture were related to the varying capacity for these communities to respond to an added stressor in the ecosystem (nutrient stress). In some sites, nutrient enrichments (at levels that are stressful) simplified already simple networks. Because our networks are built with traits that characterize ecosystem resilience generically to a wide range of stressors, this simplification could signal a loss of response capacity to future stressors under eutrophic conditions. Our findings represent a novel translation of resilience concepts from theory to the real world and this translation is critical for improving predictions of changes in ecosystem state that occur after a point when resilience capacity is lost.

Variability in nutrient treatment effect-sizes across sites was driven by where the system began from (i.e. the baseline network complexity in the control plots). The more complex the initial network (i.e. the control network), the less variability in responses to added nutrient stress; conversely, the simpler the initial network, the more variable the response (figure 4*a–d*). High levels of nutrients resulted predominantly in negative effects on networks, but the effect sizes were more variable for sites with initially simpler networks. Conversely, medium levels of nutrients sometimes had positive effects and sometimes negative effects, but similarly the variability was highest in the simpler networks. Our results suggest that the thresholds for nutrient effects are more unpredictable at sites with simpler networks, and the change in variability in nutrient effect-size along the gradient of baseline network complexity could be a real-world demonstration of ‘flickering’ which is indicative of ecosystem instability before a threshold is reached [37,38].

The network complexity-stability debate stems back to the 1950s, and early views were that more complex networks are the most stable. However, that debate has developed more nuances, with discussions of different types of stability in food web and species interaction networks [22,39]. Theoretical and empirical work has demonstrated the role of many weak interactions in promoting ecological network stability [40,41]. The stability debate also links to systems thinking on resilience. An ecosystem can resist shifts in state by either being rigid to change (i.e. stable), or by being adaptable/flexible to change (i.e. unstable but resilient) [23]. Our results link well to these abstract and conceptual resilience and stability concepts. Conceptually, rigidity of an ecological network could occur via a few very strong interactions, so even if a network is very simple, a few strong connections can stabilize the network and make it strong. However, simple networks can also be made up of few weak interactions that are brittle to break with changing conditions. This variation in the architecture of simple networks could explain the variable responses and thresholds to added stress (evidenced by the variable responses in simple networks in figure 4). While the simple rigid networks might be very strong and stable, once the few connections break, there is less opportunity for adaptation. Conversely, complex networks with many weak connections could contain a level of flexibility that allows the ecosystem to adapt and reassemble to changes (i.e. the many connections offer multiple options for responding; evidenced by the resilience of the more complex networks in figure 4).

Network complexity is in part related to species richness. In this study, the relationship between species richness and network complexity was linear to a point and then highly variable at the upper end of the richness spectrum, signalling that in more species rich systems, complexity is driven by something other than the number of species present (electronic supplementary material S2, figure S2.2). Communities with simple networks usually contain fewer species than more complex networks, but our results suggest that simple networks could potentially either contain a few highly resilient species or a few highly vulnerable ones. Compared to other soft-sediment communities, our sites encompass the middle portion of the possible diversity gradient [42] (taxa richness ranged from 19–48 taxa per site). That is, our sites are not systems dominated by large bioturbating shrimps or large structure forming macrofauna at the high diversity end of the scale, nor are they systems dominated by monocultures of capitelids at the low diversity end. Despite the constrained species gradient in our study, we were still able to demonstrate subtle shifts in the erosion of resilience that occurred without drastic species losses that characterize larger diversity gradients. Relationships between networks and response capacity would likely be different at the more degraded end of the spectrum, or across more extreme diversity gradients. In more degraded sites, network complexity may be lower, but quite stable and resilient to change given the species that are left are often those that are generalists. Conversely, in highly diverse sites (i.e. those with 100 s of species), different patterns might be seen as complexity is driven more by the specific combination of traits present and levels of redundancy.

An added strength of our study is the environmental gradients it encompasses. All of the sites are relatively undegraded and still contained the dominant shellfish species *Macomona liliana* and *Austrovenus stutchburyi*, both of which cannot occur at high densities in highly degraded estuaries (i.e. eutrophic or highly muddy [43,44]). Identifying the more subtle shifts in resilience over smaller environmental and diversity gradients informs a mechanistic understanding of what happens before tipping points. For example, research on the effects of eutrophication in estuaries is bias towards already highly degraded and eutrophic estuaries, which limits understanding of the indicators of change that occur before major shifts [31]. Further application of analysis to a wider range of environmental conditions, and linkages with functional losses across tipping points represents a future expansion of these concepts.

In addition to the broader resilience concepts that our analysis has demonstrated, we also show that resistance and recovery traits are useful for assessing the resilience potential of communities to stress. The use of traits can complement traditional community analyses, and given the changes demonstrated here, would be useful to include in monitoring programs that are focused on monitoring for changes in resilience of ecological communities. Our experiment offers a real world demonstration of the erosion of resilience capacity (with loss of response diversity) along natural environmental gradients and with an added stressor, and given that the simpler networks resulted in more variable responses to added stress, this could signal that a loss of response capacity with one environmental gradient or stressor can reduce resilience and system stability to another.

## Data Availability

The data used in this manuscript are available from the Dryad Digital Repository [36]. Additional results and figures are provided in the electronic supplementary material [45].

## References

[1] Vitousek PM. 1977 Human domination of Earth's ecosystems. Science **277**, 494-499. (10.1126/science.277.5325.494)

[2] Rockstrom J et al. 2009 A safe operating space for humanity. Nature **461**, 472-475. (10.1038/461472a) 19779433

[3] Scheffer M, Carpenter S, Foley JA, Folke C, Walker B. 2001 Catastrophic shifts in ecosystems. Nature **413**, 591-596. (10.1038/35098000) 11595939

[4] Oliver TH, Isaac NJB, August TA, Woodcock BA, Roy DB, Bullock JM. 2015 Declining resilience of ecosystem functions under biodiversity loss. Nat. Commun. **6**, 10122. (10.1038/ncomms10122) 26646209 PMC4686828

[5] Mori AS, Furukawa T, Sasaki T. 2013 Response diversity determines the resilience of ecosystems to environmental change. Biol. Rev. **88**, 349-364. (10.1111/brv.12004) 23217173

[6] Naeem S, Bunker DE, Hector A, Loreau M, Perrings C. 2009 Biodiversity, ecosystem functioning, and human wellbeing: an ecological and economic perspective. Oxford, UK: Oxford University Press.

[7] Cardinale BJ et al. 2012 Biodiversity loss and its impact on humanity. Nature **486**, 59-67. (10.1038/nature11148) 22678280

[8] Hooper DU et al. 2005 Effects of biodiversity on ecosystem functioning: a consensus of current knowledge. Ecol. Monogr. **75**, 3-35. (10.1890/04-0922)

[9] Rodil IF, Lohrer AM, Hewitt JE, Townsend M, Thrush SF, Carbines M. 2013 Tracking environmental stress gradients using three biotic integrity indices: advantages of a locally-developed traits-based approach. Ecol. Indic. **34**, 560-570. (10.1016/j.ecolind.2013.06.023)

[10] Spasojevic MJ, Copeland S, Suding KN. 2014 Using functional diversity patterns to explore metacommunity dynamics: a framework for understanding local and regional influences on community structure. Ecography **37**, 939-949. (10.1111/ecog.00711)

[11] Suding KN, Goldstein LJ. 2008 Testing the holy grail framework: using functional traits to predict ecosystem change. New Phytol. **180**, 559-562. (10.1111/j.1469-8137.2008.02650.x) 19138225

[12] Funk JL et al. 2017 Revisiting the Holy Grail: using plant functional traits to understand ecological processes. Biol. Rev. **92**, 1156-1173. (10.1111/brv.12275) 27103505

[13] Naeem S. 1998 Species redundancy and ecosystem reliability. Conserv. Biol. **12**, 39-45. (10.1111/j.1523-1739.1998.96379.x)

[14] Biggs CR et al. 2020 Does functional redundancy affect ecological stability and resilience? A review and meta-analysis. Ecosphere **11**, e03184. (10.1002/ecs2.3184)

[15] Mouillot D et al. 2014 Functional over-redundancy and high functional vulnerability in global fish faunas on tropical reefs. Proc. Natl Acad. Sci. USA **111**, 13757-13 762. (10.1073/pnas.1317625111) 25225388 PMC4183327

[16] Elmqvist T, Folke C, Nyström M, Peterson G, Bengtsson J, Walker B, Norberg J. 2003 Response diversity, ecosystem change, and resilience. Front. Ecol. Environ. **1**, 488-494. (10.1890/1540-9295(2003)001[0488:RDECAR]2.0.CO;2)

[17] Suding KN et al. 2008 Scaling environmental change through the community-level: a trait-based response-and-effect framework for plants. Glob. Change Biol. **14**, 1125-1140. (10.1111/j.1365-2486.2008.01557.x)

[18] de Juan S, Bremner J, Hewitt J, Törnroos A, Mangano MC, Thrush S, Hinz H. 2022 Biological traits approaches in benthic marine ecology: dead ends and new paths. Ecol. Evol. **12**, e9001. (10.1002/ece3.9001) 35784057 PMC9163796

[19] Mouillot D, Graham NAJ, Villéger S, Mason NWH, Bellwood DR. 2013 A functional approach reveals community responses to disturbances. Trends Ecol. Evol. **28**, 167-177. (10.1016/j.tree.2012.10.004) 23141923

[20] Mouchet MA, Villéger S, Mason NWH, Mouillot D. 2010 Functional diversity measures: an overview of their redundancy and their ability to discriminate community assembly rules. Funct. Ecol. **24**, 867-876. (10.1111/j.1365-2435.2010.01695.x)

[21] Siwicka E, Thrush SF, Hewitt JE. 2020 Linking changes in species-trait relationships and ecosystem function using a network analysis of traits. Ecol. Appl. **30**, e02010. (10.1002/eap.2010) 31556174

[22] Landi P, Minoarivelo HO, Brännström Å, Hui C, Dieckmann U. 2018 Complexity and stability of ecological networks: a review of the theory. Popul. Ecol. **60**, 319-345. (10.1007/s10144-018-0628-3)

[23] Holling CS. 1973 Resilience and stability of ecological systems. Annu. Rev. Ecol. Syst. **4**, 1-23. (10.1146/annurev.es.04.110173.000245)

[24] Thrush SF, Hewitt JE, Cummings VJ, Ellis JI, Hatton C, Lohrer A, Norkko A. 2004 Muddy waters: elevating sediment input to coastal and estuarine habitats. Front. Ecol. Environ. **2**, 299-306. (10.1890/1540-9295(2004)002[0299:MWESIT]2.0.CO;2)

[25] Thrush SF et al. 2021 Cumulative stressors reduce the self-regulating capacity of coastal ecosystems. Ecol. Appl. **31**, e02223. (10.1002/eap.2223) 32869444 PMC7816261

[26] Gammal J et al. 2022 Stressors increase the impacts of coastal macrofauna biodiversity loss on ecosystem multifunctionality. Ecosystems 1-14. (10.1007/s10021-022-00775-4)

[27] Douglas EJ, Pilditch CA, Hines LV, Kraan C, Thrush SF. 2016 In situ soft sediment nutrient enrichment: a unified approach to eutrophication field experiments. Mar. Pollut. Bull. **111**, 287-294. (10.1016/j.marpolbul.2016.06.096) 27389457

[28] Douglas EJ, Pilditch CA, Kraan C, Schipper LA, Lohrer AM, Thrush SF. 2017 Macrofaunal functional diversity provides resilience to nutrient enrichment in coastal sediments. Ecosystems. **20**, 1324-1336. (10.1007/s10021-017-0113-4)

[29] Mangan S, Lohrer AM, Thrush SF, Pilditch CA. 2020 Water column turbidity not sediment nutrient enrichment moderates microphytobenthic primary production. J. Mar. Sci. Eng. **8**, 732. (10.3390/jmse8100732)

[30] Mangan S, Bryan KR, Thrush SF, Gladstone-Gallagher RV, Lohrer AM, Pilditch CA. 2020 Shady business: the darkening of estuaries constrains benthic ecosystem function. Mar. Ecol. Prog. Ser. **647**, 33-48. (10.3354/meps13410)

[31] Vieillard AM, Newell SE, Thrush S. 2020 Recovering from Bias: a call for further study of under-represented tropical and low-nutrient estuaries. J. Geophys. Res. Biogeosci. **125**, e2020JG005766. (10.1029/2020JG005766)

[32] Arar EJ, Collins GB. 1997 Method 445.0: In vitro determination of chlorophyll a and pheophytin a in marine and freshwater algae by fluorescence. Cincinnati, OH: US Environmental Protection Agency.

[33] Gladstone-Gallagher RV, Hewitt JE, Thrush SF, Brustolin MC, Villnas A, Valanko S, Norkko A. 2021 Identifying ‘vital attributes’ for assessing disturbance-recovery potential of seafloor communities. Ecol. Evol. **11**, 6091-6103. (10.1002/ece3.7420) 34141205 PMC8207434

[34] Griffith DM, Veech JA, Marsh CJ. 2016 cooccur: probabilistic species co-occurrence analysis in R. J. Stat. Softw. **69**, 1-17. (10.18637/jss.v069.c02)

[35] Delmas E et al. 2019 Analysing ecological networks of species interactions. Biol. Rev. **94**, 16-36. (10.1111/brv.12433) 29923657

[36] Thrush S. 2023 Data from: Cumulative stressors reduce the self-regulating capacity of coastal ecosystems. *Dryad Digital Repoistory*. (10.5061/dryad.8cz8w9gms2020) PMC7816261 32869444

[37] Dakos V, van Nes EH, Scheffer M. 2013 Flickering as an early warning signal. Theor. Ecol. **6**, 309-317. (10.1007/s12080-013-0186-4)

[38] Seekell DA et al. 2011 Conditional heteroscedasticity as a leading indicator of ecological regime shifts. Am. Nat. **178**, 442-451. (10.1086/661898) 21956023

[39] Namba T. 2015 Multi-faceted approaches toward unravelling complex ecological networks. Popul. Ecol. **57**, 3-19. (10.1007/s10144-015-0482-5)

[40] Ings TC et al. 2009 Review: ecological networks – beyond food webs. J. Anim. Ecol. **78**, 253-269. (10.1111/j.1365-2656.2008.01460.x) 19120606

[41] Berlow EL. 1999 Strong effects of weak interactions in ecological communities. Nature **398**, 330-334. (10.1038/18672)

[42] Pearson T, Rosenberg R. 1978 Macrobenthic succession in relation to organic enrichment and pollution of the marine environment. Oceanogr. Mar. Biol.: Annu. Rev. **16**, 229-311.

[43] Thrush SF, Hewitt J, Norkko A, Nicholls PE, Funnell GA, Ellis JI. 2003 Habitat change in estuaries: predicting broad-scale responses of intertidal macrofauna to sediment mud content. Mar. Ecol. Prog. Ser. **263**, 101-112. (10.3354/meps263101)

[44] Ellis JI, Clark D, Atalah J, Jiang W, Taiapa C, Patterson M, Sinner J, Hewitt J. 2017 Multiple stressor effects on marine infauna: responses of estuarine taxa and functional traits to sedimentation, nutrient and metal loading. Sci. Rep. **7**, 12013. (10.1038/s41598-017-12323-5) 28931887 PMC5607226

[45] Gladstone-Gallagher RV, Hewitt JE, Siwicka E, Gammal JM, Brustolin MC, Norkko A, Pilditch CA, Thrush SF. 2023 Ecological network analysis of traits reveals variable response capacity to stress. Figshare. (10.6084/m9.figshare.c.6602687) PMC10154921 37132238

